# Sex, Scavengers, and Chaperones: Transcriptome Secrets of Divergent *Symbiodinium* Thermal Tolerances

**DOI:** 10.1093/molbev/msw119

**Published:** 2016-06-14

**Authors:** Rachel A. Levin, Victor H. Beltran, Ross Hill, Staffan Kjelleberg, Diane McDougald, Peter D. Steinberg, Madeleine J. H. van Oppen

**Affiliations:** ^1^Centre for Marine Bio-Innovation, The University of New South Wales, Sydney, NSW, Australia; ^2^School of Biological Earth and Environmental Sciences, The University of New South Wales, Sydney, NSW, Australia; ^3^Australian Institute of Marine Science, Townsville MC, QLD, Australia; ^4^Macquarie University, Sydney, NSW, Australia; ^5^Singapore Centre on Environmental Life Sciences Engineering, Nanyang Technological University, Singapore; ^6^The iThree Institute, University of Technology Sydney, Sydney, NSW, Australia; ^7^Sydney Institute of Marine Science, Mosman, NSW, Australia; ^8^School of BioSciences, The University of Melbourne, Parkville, VIC, Australia

**Keywords:** symbiodinium, dinoflagellate, thermal tolerance, acclimation, coral, bleaching, transcriptomics.

## Abstract

Corals rely on photosynthesis by their endosymbiotic dinoflagellates (*Symbiodinium* spp.) to form the basis of tropical coral reefs. High sea surface temperatures driven by climate change can trigger the loss of *Symbiodinium* from corals (coral bleaching), leading to declines in coral health. Different putative species (genetically distinct types) as well as conspecific populations of *Symbiodinium* can confer differing levels of thermal tolerance to their coral host, but the genes that govern dinoflagellate thermal tolerance are unknown. Here we show physiological and transcriptional responses to heat stress by a thermo-sensitive (physiologically susceptible at 32 °C) type C1 *Symbiodinium* population and a thermo-tolerant (physiologically healthy at 32 °C) type C1 *Symbiodinium* population. After nine days at 32 °C, neither population exhibited physiological stress, but both displayed up-regulation of meiosis genes by ≥ 4-fold and enrichment of meiosis functional gene groups, which promote adaptation. After 13 days at 32 °C, the thermo-sensitive population suffered a significant decrease in photosynthetic efficiency and increase in reactive oxygen species (ROS) leakage from its cells, whereas the thermo-tolerant population showed no signs of physiological stress. Correspondingly, only the thermo-tolerant population demonstrated up-regulation of a range of ROS scavenging and molecular chaperone genes by ≥ 4-fold and enrichment of ROS scavenging and protein-folding functional gene groups. The physiological and transcriptional responses of the *Symbiodinium* populations to heat stress directly correlate with the bleaching susceptibilities of corals that harbored these same *Symbiodinium* populations. Thus, our study provides novel, foundational insights into the molecular basis of dinoflagellate thermal tolerance and coral bleaching.

## Introduction

Corals and their dinoflagellate endophotosymbionts of the genus *Symbiodinium* create the foundation of tropical coral reefs, which support hundreds of thousands of plant and animal species (Reaka-Kudla et al. 1996). Tropical reef-building corals require metabolites provided by *Symbiodinium* for their nutrition and high rates of calcification (Muscatine and Porter 1977; Barnes and Chalker 1990; Gordon and Leggat 2010). Efficient recycling of nutrients between *Symbiodinium* and corals allows entire ecosystems to flourish in low nutrient waters (Roth 2014). Rising sea surface temperatures due to climate change cause the breakdown of the *Symbiodinium*-coral symbiosis resulting in the loss of *Symbiodinium* from the coral host (i.e., coral bleaching) and, consequently, drastic declines in coral health and cover worldwide (Hoegh-Guldberg 1999; Hoegh-Guldberg et al. 2007). Climate change impact models predict that many coral reefs will be irreversibly damaged in a matter of decades (Carpenter et al. 2008; Pandolfi et al. 2011). While the exact mechanistic role that *Symbiodinium* plays in coral bleaching has yet to be uncovered, increased production of ROS, such as superoxide and hydrogen peroxide, by *Symbiodinium* cells in response to heat stress is considered to be a key factor (Suggett et al. 2008; McGinty et al. 2012). Leakage of excess ROS from *Symbiodinium* cells when inside the coral tissues (*in hospite*) may exacerbate stress-induced oxidative damage of coral tissues and lead to *Symbiodinium* expulsion (Downs et al. 2002; Krueger et al. 2015).

The genus *Symbiodinium* is highly diverse, and substantial physiological differences exist among and even within “types”, i.e., genetic variants typically designated by the nuclear ribosomal DNA internal transcribed spacer 2 (ITS2) to notionally represent species (Arif et al. 2014). Different *Symbiodinium* can strongly influence coral gene expression and bleaching susceptibility (DeSalvo et al. 2010; Oliver and Palumbi 2011; Howells et al. 2012; Yuyama et al. 2012), and it is generally thought that *Symbiodinium* are more vulnerable to heat stress than their coral host (Fitt et al. 2001). Unraveling the molecular basis of variation in *Symbiodinium* thermal tolerance is thus an essential step required to understand variation in coral bleaching susceptibility.

Although *Symbiodinium* physiological responses to heat stress are well studied (Warner et al. 1999; Tchernov et al. 2004; Suggett et al. 2008; Howells et al. 2012; McGinty et al. 2012), the underlying gene regulation is still unresolved. Much of the evidence to date suggests that *Symbiodinium* lack a transcriptional response to heat stress (Leggat et al. 2011; Putnam et al. 2013; Barshis et al. 2014; Krueger et al. 2015), which contradicts the strong evidence in other organisms that physiological changes are largely driven by regulation of mRNA synthesis and degradation (Arbeitman et al. 2002; Wilusz and Wilusz 2004; Rossouw et al. 2009; Harb et al. 2010). In *Symbiodinium*, translational regulation and post-translational modifications have been hypothesized to primarily drive changes in the proteome under heat stress (Barshis et al. 2014), as only a small collection of transcription factors have been identified in the transcriptome and genome of *Symbiodinium* (Bayer et al. 2012; Shoguchi et al. 2013). *Symbiodinium* transcriptomes have also been found to contain microRNAs (Baumgarten et al. 2013), molecules that repress translation of mRNA into proteins as well as direct and accelerate mRNA degradation (Valencia-Sanchez et al. 2006; Wu et al. 2006). Regulation of mRNA abundance may, therefore, be an important contributor to physiological responses by *Symbiodinium*.

Several previous gene expression studies in *Symbiodinium* have applied acute heat stress on the scale of hours to a few days (Baumgarten et al. 2013; Barshis et al. 2014; Rosic et al. 2014; Krueger et al. 2015), but a study on mRNA stability in the dinoflagellate *Karenia brevis* found dinoflagellate mRNA half-lives to be considerably longer than in other organisms (Morey and Van Dolah 2013). The majority of transcripts involved in the stress response, metabolism, and transcriptional regulation had half-lives over 24 h, and in some cases over four days (e.g., catalase/peroxidase, thioredoxin, and chaperone protein DnaJ) (Morey and Van Dolah 2013). Thus, some dinoflagellate genes may simply require longer periods of time to develop significant, detectable mRNA expression changes. However, Morey and Van Dolah (2013) did not measure mRNA half-lives under temperature stress, which can significantly alter mRNA stability (Castells-Roca et al. 2011; Chiba et al. 2013).

In this study, we used two heterogeneous populations of type C1 *Symbiodinium*, an ecologically important and globally distributed type associated with a diverse range of coral species (LaJeunesse et al. 2004; LaJeunesse 2005; Tonk et al. 2013). Despite having identical ITS1 and ITS2 sequences, the populations exhibit different thermal tolerances. Physiological and transcriptional analyses were conducted for each population at ambient (27 °C) and elevated (32 °C) temperatures in culture in order to investigate the molecular basis of *Symbiodinium* thermal tolerance. The populations were originally isolated from the coral *Acropora tenuis* at two separate sites on the Great Barrier Reef: South Molle Island (SM; 20°16′33″S, 148°49′36″E) and Magnetic Island (MI; 19°9′6″S, 146°51′56″E) that have average summer daily maximums of 28.2 °C and 30.1 °C, respectively. Corals harboring the thermo-sensitive SM population were previously shown to bleach after 11 days at 32 °C, whereas corals harboring the thermo-tolerant MI population remained unaffected (Howells et al. 2012). A significant reduction in photosynthetic capacity due to heat stress, a diagnostic trait of *Symbiodinium* thermal sensitivity and coral bleaching (Warner et al. 1999), accompanied loss of the SM population from its coral host at 32 °C (Howells et al. 2012). The susceptibility of each population to elevated temperature *in hospite* correlated with thermal tolerance in culture (Howells et al. 2012).

Here we report on thousands of differentially expressed genes (DEGs) in both populations exposed to elevated temperature (32 °C) that align with physiological responses. Our findings demonstrate how distinct transcriptomic plasticity and regulation of hallmark thermal tolerance genes and functional gene groups (i.e., gene ontology categories) can allow allopatric, conspecific *Symbiodinium* populations to exhibit contrasting thermal tolerances.

## Results and Discussion

### Physiological Responses of *Symbiodinium* to Heat Stress

**Fig. 1:**
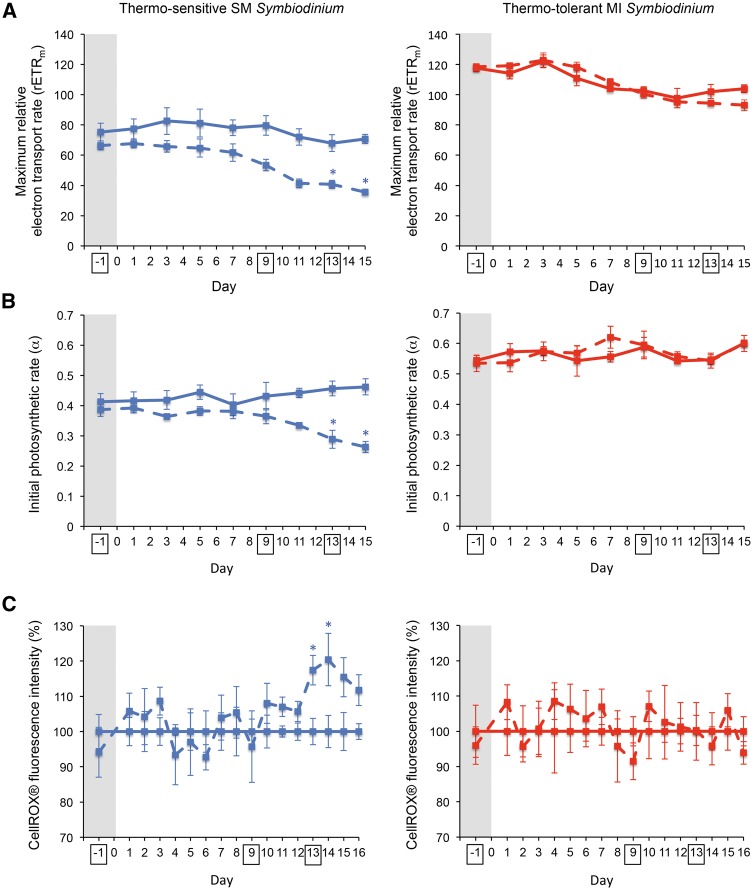
Physiological detection of *Symbiodinium* heat stress. Intact lines represent the 27 °C temperature treatment, and dashed lines represent the 32 °C temperature treatment. Before heating, all samples were kept at 27 °C (values in the grey regions). (*A*) rETRm (mean ± sem, *n* = 4). (*B*) α (mean ± sem, *n* = 4). (*C*) Leakage of ROS out of cells (mean ± sem, *n* = 4); unitless fluorescence intensities of CellROX® Orange reagent for oxidative stress detection were normalized across days by setting the fluorescence intensities of the 27 °C samples to 100%. Asterisks indicate statistically significant (PERMANOVA) differences between temperature treatments at *P* < 0.05. Sampling time points for transcriptomics are boxed. Additional physiological measurements are shown in supplementary fig. S1, Supplementary Material online.

Each population was cultured at 27 °C and 32 °C in two replicate incubators (supplementary table S1, Supplementary Material online) to avoid potential artifacts from individual incubators in our results. Physiological measurements for detection of cellular heat stress were used to determine sampling time points for transcriptomics that were anticipated to identify DEGs between temperature treatments (fig. 1*A*–*C* and supplementary fig. S1*A–D*, Supplementary Material online). On day 13, both the maximum relative electron transport rate for photosynthesis (rETRm) and initial photosynthetic rate (α) significantly decreased (*P* < 0.05) at 32 °C compared with 27 °C in the SM population only (fig. 1*A* and *B*). Decreased photosynthetic ability of *Symbiodinium* has been strongly connected to *Symbiodinium* thermal sensitivity and coral bleaching susceptibility (Warner et al. 1999; Takahashi et al. 2009; Ragni et al. 2010; Howells et al. 2012). Additionally, a significant increase (*P* < 0.05) in general ROS leakage out of *Symbiodinium* cells was detected in the SM population at 32 °C beginning on day 13 (fig. 1*C*), an observation that is consistent with evidence that coral bleaching is largely driven by increased ROS inside coral tissues (Downs et al. 2002; Suggett et al. 2008). Therefore, day 13 was chosen as a sampling time point for transcriptomics, along with day −1 to account for any pre-experimental DEGs between groups. Day 9, the potential start of the declining trend in rETRm in the SM population, was also selected as a sampling time point for transcriptomics to determine if the transcriptional response to heat stress precedes significant physiological damage. The overall lower photosynthetic efficiency of the SM population may be due to the lower amounts of photosynthetic pigments (chlorophyll *a* and β−carotene) in cells from the SM population compared to those from the MI population (Howells et al. 2012).

### Plasticity of *Symbiodinium* Transcriptomes under Heat Stress

The *de novo* assembled transcriptomes from the SM and MI populations were composed of 106,097 and 93,377 putative genes, respectively. However, the number of genes in each transcriptome likely overestimates the number of genes expressed by a single genotype because our study used heterogeneous populations rather than clonal cultures. Each population consisted of an unknown diversity of individuals within type C1 and, therefore, an unknown diversity of transcript variants and alleles. The SM and MI populations, rather than clonal cultures, were chosen in our study as their bleaching responses at 32 °C have been characterized *in hospite* (Howells et al. 2012) and as heterogeneous populations are more representative of symbiont communities inhabiting Great Barrier Reef corals. Average transcript lengths (SM: 858.1 bp and MI: 911.4 bp; supplementary table S2, Supplementary Material online) for the SM and MI transcriptomes were in range with those for previously published *Symbiodinium* transcriptomes (Bayer et al. 2012; Parkinson et al. 2016). Quantitative assessment of conserved eukaryotic orthologs (Simão et al. 2015) revealed that the SM and MI transcriptomes are the most complete *Symbiodinium* transcriptomes of the publicly accessible, published *Symbiodinium* transcriptomes to date (Bayer et al. 2012; Ladner et al. 2012; Baumgarten et al. 2013; Rosic et al. 2015; Xiang et al. 2015; Parkinson et al. 2016) (supplementary table S3, Supplementary Material online). The biological coefficient of variance (BCV) for gene expression across replicates in each population was found to be < 0.2 on all time points, well below the commonly accepted variance threshold of 0.4 (McCarthy et al. 2012; Chen et al. 2014).

For differential gene expression analysis, we defined significant biological relevance as ≥ 4-fold differential expression between temperature treatments combined with a conservative false discovery rate (FDR) ≤ 0.001. On day −1 prior to heat treatment, only one DEG (TR83958|c0_g1, a putative 10 kDa chaperonin) in the SM population and no DEGs in the MI population were found between the experimental groups of each population that had been pre-assigned to the different temperature treatments. TR83958|c0_g1 from the SM population was not differentially expressed on either of the later time points. The lack of DEGs between experimental groups in both populations before heating corroborates that DEGs detected on days 9 and 13 were in response to the temperature treatment and that differential expression cutoffs (fold ≥ 4 and FDR ≤ 0.001 between temperature treatments) and replication (*n* = 4) were adequate to achieve a high signal to noise ratio.

**Fig. 2:**
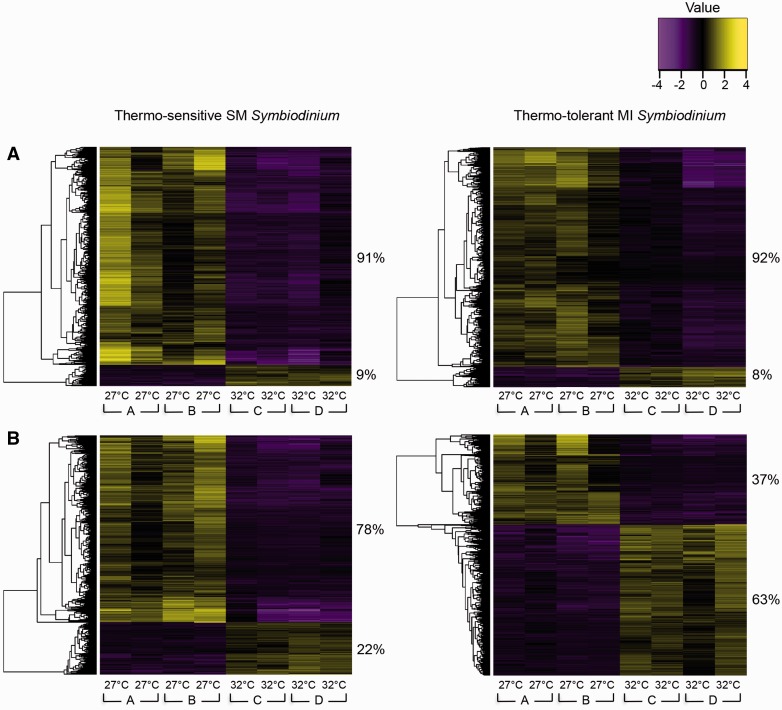
Hierarchical clustering of DEGs. Heat maps show genes (rows) with differential expression (Trinity/edgeR: fold ≥ 4, FDR ≤ 0.001) between 27 °C and 32 °C samples (columns) for each population on (*A*) day 9 and (*B*) day 13. Expression values (fpkm) are log_2_-transformed and then median-centered by gene. Heat map values were calculated by subtracting each gene’s median log_2_(fpkm) value from its log_2_(fpkm) value in each sample. The proportions (%) of DEGs that were up- or down-regulated due to heat stress are noted to the right of the two main gene clusters of each heat map. Genes are independently clustered for each population at each time point. Samples from replicate cultures at each temperature treatment are presented in the same order for each time point. The experimental incubator (A, B, C, or D) that housed each sample is noted below the temperature treatment.

On day 9, a total of 4,608 and 2,379 DEGs were identified between the temperature treatments in the SM and MI populations, respectively. The vast majority of DEGs in the SM population (4,199 or 91%) and MI population (2,179 or 92%) were down-regulated at 32 °C relative to expression levels at 27 °C (fig. 2*A*). Down-regulation of the majority of DEGs in response to elevated temperature has been previously observed in marine organisms including *Symbiodinium* and corals (Baumgarten et al. 2013; Yampolsky et al. 2014; Bay and Palumbi 2015) and may be a strategy to conserve energy when confronted with environmental stress (Yampolsky et al. 2014).

On day 13, a total of 4,272 and 3,513 DEGs were identified between the temperature treatments in the SM and MI populations, respectively. The SM population responded similarly to 32 °C on day 13 as on day 9 by down-regulating the majority of DEGs (3,341 or 78%). Conversely, the MI population up-regulated the majority of DEGs (2,201 or 63%) at 32 °C, suggesting acclimation to 32 °C (fig. 2*B*). Our results demonstrate that some *Symbiodinium* do exhibit transcriptomic plasticity and are capable of up-regulating a large number of genes in response to elevated temperature.

In the SM population at 32 °C, 239 and 1,925 genes remained up- and down-regulated, respectively, on both days 9 and 13. In the MI population at 32 °C, 113 and 585 genes remained up- and down-regulated, respectively, on both days 9 and 13. Interestingly, 353 genes in the MI population at 32 °C that were down-regulated on day 9 became up-regulated on day 13, whereas no genes switched from down- to up-regulation in the SM population at 32 °C. No up-regulated genes on day 9 became down-regulated on day 13 in either population at 32 °C.

### Gene Ontology (GO) Analysis of DEGs to Identify Functional Gene Groups Involved in Thermal Tolerance

**Fig. 3:**
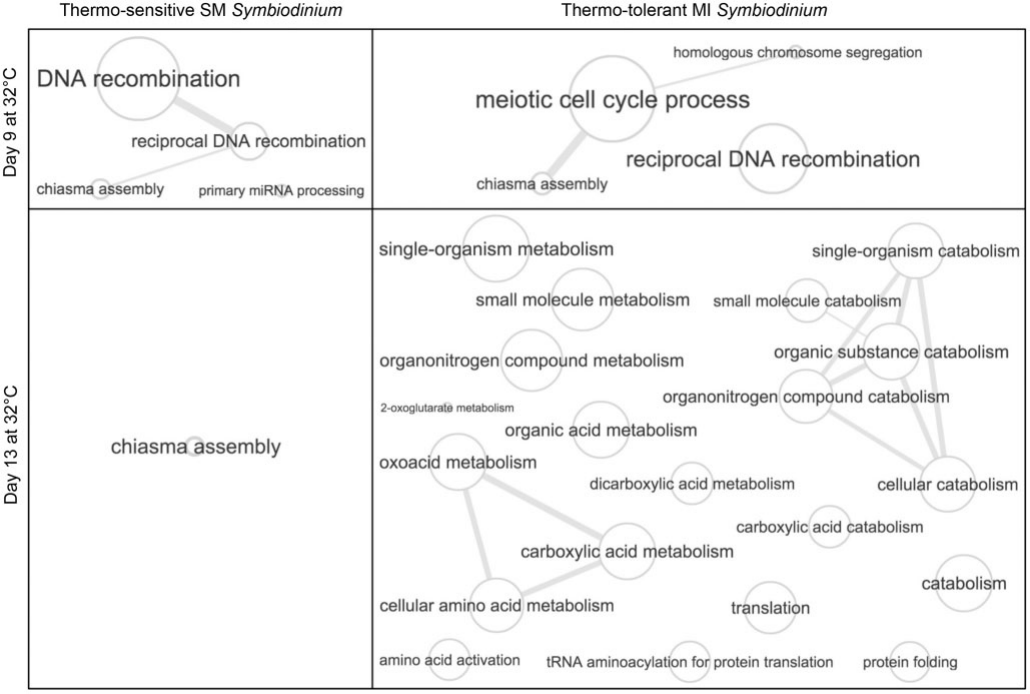
Unsuccessful versus successful acclimation to elevated temperature. GO relationship graphs for enriched biological process GO categories (Goseq: FDR < 0.05) at 32 °C were generated using REVIGO (Supek et al. 2011). Bubble size indicates the frequency of the GO category in the UniProt database relative to the other GO categories that are in the same section. Lines link similar GO categories, and the line width indicates the degree of similarity between the GO categories relative to others in the same section. Redundant GO categories (similarity > 0.9) were collapsed into the category that is most frequent in the UniProt database. Graphs for enriched molecular function GO categories (e.g., unfolded protein binding and glutamate dehydrogenase activity) and enriched cellular component GO categories (e.g., oxidoreductase complex and motile cilium) at 32 °C are not included in this figure. Full GO analysis results are listed in supplementary dataset S1*A*–*M*, Supplementary Material online.

GO analysis (FDR < 0.05) of genes at 32 °C further supported that only the MI population acclimated to elevated temperature (fig. 3 and supplementary dataset S1*A*–*M*, Supplementary Material online), consistent with only the SM population suffering physiological damage after 13 days of heat stress. Acclimation to stressful conditions through transcriptional changes has been observed in other marine organisms including corals (Nymark et al. 2009; Yampolsky et al. 2014; Bay and Palumbi 2015; Hennon et al. 2015), but never before in *Symbiodinium*, or to our knowledge, in any dinoflagellate species.

On day 9, the down-regulated genes in the SM population at 32 °C were enriched for 133 GO categories consisting of 26 metabolic and biosynthetic categories, whereas the down-regulated genes in the MI population at 32 °C were enriched for 311 GO categories that included 45 metabolic and biosynthetic categories (supplementary dataset S1*B* and *D*, Supplementary Material online). Reduced metabolic and biosynthetic activity has been shown to correlate with increased survival time of organisms under stress, as it allows for substantial energetic savings (Hand and Hardewig 1996). Specifically in the case of heat stress, such metabolic compensation is considered an acclimatory mechanism to elevated temperature in the zooplankton *Daphnia pulex* (Yampolsky et al. 2014).

The small number of significantly up-regulated genes in the SM and MI populations at 32 °C on day 9 was enriched for six and seven GO categories, respectively (fig. 3 and supplementary dataset S1*A* and *C*, Supplementary Material online). The majority of enriched GO categories in both populations were specific to meiosis, suggesting that *Symbiodinium* cells were participating in sexual rather than strictly asexual reproduction under heat stress. Potential sexual reproduction by *Symbiodinium* is particularly noteworthy since meiosis creates genetic diversity through chromosomal modifications and recombination, therefore promoting adaptation (Tamburini and Tyler 2005; D'Souza and Michiels 2010; Becks and Agrawal 2012). Meiosis-specific genes have been previously identified in *Symbiodinium* (Chi et al. 2014; Rosic et al. 2015), but so far, sexual reproduction has not been directly observed. However, recent studies have hypothesized that recombination during meiosis may be a mechanism of adaptation in *Symbiodinium* (Chi et al. 2014; Wilkinson et al. 2015). Other dinoflagellate species can rapidly increase genetic diversity by switching from mitosis to meiosis and can enter a sexual cyst life cycle stage when exposed to stressful conditions in order to survive and adapt (Figueroa et al. 2010; Bravo and Figueroa 2014), though no visually apparent *Symbiodinium* cysts were observed in our study.

On day 13, the dramatic increase in up-regulated genes in the MI population at 32 °C was characterized by enrichment of 60 GO categories (fig. 3 and supplementary dataset S1*G*, Supplementary Material online), which included nine metabolic categories along with several of the corresponding catabolic categories for maintaining cellular homeostasis. Importantly, GO categories for unfolded protein binding, protein folding, glutamate dehydrogenase (NAD+) activity and the oxidoreducatase complex—all of which are involved in stress tolerance (Srivastava and Singh 1987; Singh and Grover 2008; Bita and Gerats 2013; Tercé-Laforgue et al. 2015)—also became enriched in the up-regulated genes in the MI population at 32 °C. The subset of 353 up-regulated genes at 32 °C, which had been down-regulated at 32 °C on day 9, was enriched for 29 GO categories including seven for metabolism and biosynthesis, one for oxidoreductase activity, and one for motile cilium (supplementary dataset S1*M*, Supplementary Material online). The down-regulated genes in the MI population at 32 °C on day 13 were enriched for 160 GO categories covering a broad array of processes, though only 13 metabolic and biosynthetic GO categories were present (supplementary dataset S1*H*, Supplementary Material online). The largely reduced metabolic compensation at 32 °C on day 13 (down-regulated genes enriched for 13 metabolic and biosynthetic GO categories) relative to day 9 (down-regulated genes enriched for 45 metabolic and biosynthetic GO categories), along with up-regulation of genes enriched for metabolic and stress tolerance GO categories on day 13, suggests that the MI population had acclimated to 32 °C.

In contrast, GO enrichment analysis indicated that the SM population was unable to acclimate to 32 °C by day 13. The down-regulated genes in the SM population at 32 °C on day 13 were enriched for 135 GO categories, including 19 for metabolism and biosynthesis and one for the oxidoreductase complex (supplementary dataset S1*F*, Supplementary Material online). Only three GO categories were enriched in the up-regulated genes in the SM population at 32 °C, one of which was the meiosis GO category, chiasma assembly, potentially signifying a continued attempt to adapt by producing genetic diversity through sexual recombination (fig. 3 and supplementary dataset S1*E*, Supplementary Material online). No metabolic, biosynthetic, or stress tolerance GO categories became enriched in the up-regulated genes at 32 °C. The extended duration of metabolic compensation experienced by the SM population compared with the MI population under heat stress could potentially cause starvation of its coral host, which may contribute to the higher bleaching susceptibility of corals harboring the SM population (Howells et al. 2012).

### Regulation of Hallmark Genes Involved in Adaptation and Thermal Tolerance

**Fig. 4:**
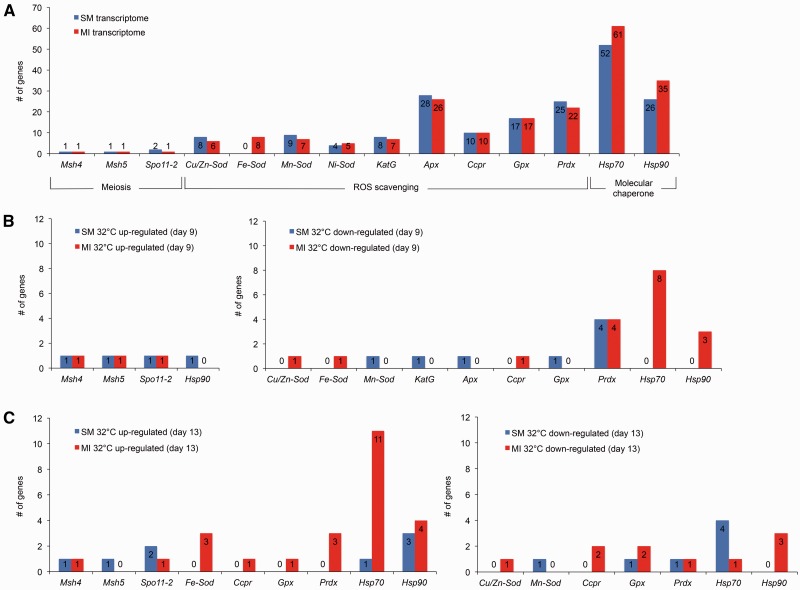
Regulation of meiosis, ROS scavenging, and molecular chaperone genes. (*A*) The number of genes for gene types involved in sexual reproduction or thermal tolerance in the SM and MI transcriptomes. The number of DEGs at 32 °C from each gene type (Trinity/edgeR: fold ≥ 4 and FDR ≤ 0.001 relative to 27 °C) are shown for each population on (*B*) day 9 and (*C*) day 13. Gene types that had no DEGs in either population are excluded from (*B*) and (*C*). Gene abbreviations are as follows: mutS protein homolog 4 (*Msh4*), mutS protein homolog 5 (*Msh5*), meiotic recombination protein Spo11-2 (*Spo11-2*), copper/zinc superoxide dismutase (*Cu/Zn-Sod*), iron superoxide dismutase (*Fe-Sod*), manganese superoxide dismutase (*Mn-Sod*), nickel superoxide dismutase (*Ni-Sod*), catalase-peroxidase (*KatG*), ascorbate peroxidase (*Apx*), cytochrome *c* peroxidase (*Ccpr*), glutathione peroxidase (*Gpx*), peroxiredoxin (*Prdx*), heat shock protein 70 (*Hsp70*), heat shock protein 90 (*Hsp90*). Additional genes are shown in supplementary fig. S2, Supplementary Material online. DEG annotation and differential expression details are provided in supplementary tables S4–S7, Supplementary Material online.

Although the responses of the two *Symbiodinium* populations to heat stress differed, both transcriptomes contained comparable suites of meiosis-specific and thermal tolerance genes (fig. 4*A* and supplementary fig. S2*A*, Supplementary Material online) that are consistent with gene content found in other *Symbiodinium* (Bayer et al. 2012; Chi et al. 2014; Krueger et al. 2015; Rosic et al. 2015). However, a striking difference in gene content between the transcriptomes of the SM and MI populations was the expression of eight iron superoxide dismutase (*Fe-Sod*) genes in the MI transcriptome, whereas no *Fe-Sod* genes were expressed in the SM transcriptome, implying that these genes are either absent from the SM population or their expression is suppressed through epigenetic regulation. Detectable *Fe-Sod* gene expression is inconsistent among other *Symbiodinium* (Krueger et al. 2015), and phylogenetic evidence that the acquisition of several ROS scavenging genes by *Symbiodinium* has resulted from horizontal gene transfer (Krueger et al. 2015) indicates that some *Symbiodinium* genomes may lack *Fe-Sod* genes entirely. Successful PCR amplification of the most highly expressed *Fe-Sod* gene (TR20255|c0_g1, open reading frame: 674-78[-]) from the genomic DNA of the MI population but not the SM population highlights the robustness of our transcriptome assemblies and supports that some gene content varies between the populations (supplementary fig. S3*A*, Supplementary Material online). However, our PCR results cannot confirm that no *Fe-Sod* genes are present in the SM population, as the primers were specific to the open reading frame of TR20255|c0_g1. Differences in nucleotide sequence between the open reading frame of TR20255|c0_g1 and the other seven *Fe-Sod* genes in the MI population, as well as the *Fe-Sod* genes identified by Krueger et al. (2015) in types B1, E, and F1 *Symbiodinium* (supplementary fig. S3*B*, Supplementary Material online), suggest that alternative *Fe-Sod* genes could be in the SM population but be silenced or expressed below the detectable level. Though, it should also be noted that Krueger et al. (2015) failed to find any *Fe-Sod* genes expressed in types C1, C3, C15, and D *Symbiodinium.*

On days 9 and 13, both populations maintained up-regulation of meiosis-specific genes at 32 °C (fig. 4*B* and *C*), although statistically significant up-regulation (fold ≥ 4, FDR ≤ 0.001) of mutS homolog 5 (*Msh5*) was limited to day 9 in the MI population. The heterodimer partners mutS homolog 4 (*Msh4*) and *Msh5* are members of the *Msh* gene family. Unlike the other *Msh* genes that are involved in mismatch repair, DNA damage repair, and mitotic recombination (Modrich and Lahue 1996; Wang and Qin 2003; Stojic et al. 2004), studies in a wide range of organisms (including humans, mice, yeast, *Caenorhabditis elegans, Arabidopsis thaliana*, and *Tetrahymena thermophile*) show that *Msh4* and *Msh5* genes are essential and specific to meiosis (Hollingsworth et al. 1995; Bocker et al. 1999; Kelly et al. 2000; Kneitz et al. 2000; Novak et al. 2001; Argueso et al. 2004; Higgins et al. 2004; Shodhan et al. 2014). MSH4 and MSH5 proteins form a meiosis-specific sliding clamp that holds and pairs homologous chromosomes during meiosis (Kneitz et al. 2000; Snowden et al. 2004). Mutations to *Msh4* and *Msh5* genes have both been shown to affect crossing over of homologous chromosomes but not to affect mismatch repair (Ross-Macdonald and Roeder 1994; Hollingsworth et al. 1995). However, some studies indicate that MSH4 and/or MSH5 proteins may have additional functions outside of meiosis processes such as DNA damage response (Her et al. 2003; Sekine et al. 2007; Tompkins et al. 2009). To consider whether *Symbiodinium Msh4* and *Msh5* genes may atypically function in non-meiotic pathways with other *Msh* genes, we investigated the gene expression of *Msh*1, 2, 3, and 6 in the *Symbiodinium* transcriptomes. Interestingly, none of the *Msh* genes besides *Msh4* and *Msh5* were up-regulated at 32 °C, supporting that meiosis-specific processes were induced rather than mismatch repair, DNA damage repair, or mitotic recombination.

The meiotic recombination protein Spo11-2 (*Spo11-2*) gene was also up-regulated in both *Symbiodinium* transcriptomes on days 9 and 13 at 32 °C. *Spo11-2* and its paralog, the meiotic recombination protein Spo11-1 (*Spo11-1*) gene, are the meiosis-specific members of the *Spo11* gene family. SPO11-1 and SPO11-2 proteins create meiosis-specific double-strand breaks in DNA and form the synaptonemal complex to initiate meiosis (Cao et al. 1990; Keeney et al. 1997; Tsubouchi and Roeder 2005; Keeney 2007). Mutations to *Spo11-2* in *Arabidopsis thaliana* cause sterility and aneuploidy (Stacey et al. 2006; Hartung et al. 2007). Although we are not aware of any examples in which the *Spo11-2* gene acts outside of meiosis, future detailed studies will be important to confirm that the meiosis-specific functions of *Spo11-2*, as well as *Msh4* and *Msh5*, are conserved in *Symbiodinium*.

Superoxide dismutases are key scavengers of superoxide, peroxidases are engaged in the removal of hydrogen peroxide, and molecular chaperones are essential for refolding damaged proteins (Vierling 1991; Gill and Tuteja 2010)—making them key contributors to thermal tolerance. Despite the many examples that up-regulation of these genes confers thermal tolerance in numerous photosynthetic species (Van Breusegem et al. 1999; Tang et al. 2006; Singh and Grover 2008; Bita and Gerats 2013), many studies report no notable differential expression of these genes in *Symbiodinium* at elevated temperature (Leggat et al. 2011; Putnam et al. 2013; Barshis et al. 2014; Krueger et al. 2015). Yet, limited evidence suggests that the transcriptional heat stress response of *Symbiodinium* may involve up-regulation of some genes classically associated with thermal tolerance. The first study demonstrated through qPCR that cytochrome P450 (*Cyp450*) gene expression by type C3 *Symbiodinium* increased at 26 °C and 29 °C compared with 23–24 °C, whereas exposure to 32 °C resulted in decreased *Cyp450* expression (Rosic et al. 2010). The next study also used qPCR and showed that heat shock protein 70 (*Hsp70*) expression in type C1 *Symbiodinium* was slightly increased at approximately 30 °C, but down-regulation of *Hsp70* occurred at 32 °C (Rosic et al. 2011). An RNA-seq study of type A1 *Symbiodinium* found up-regulation of one peroxiredoxin (*Prdx*) gene, one *Hsp* gene, and one chaperone protein DnaJ (*DnaJ)* gene from exposure to 34 °C for 12 h (Baumgarten et al. 2013). However, the importance of the differential gene expression at this extreme temperature was not substantiated by sample replication, correspondence to a physiological heat stress response, or relation to a coral bleaching response (Baumgarten et al. 2013). Finally, a recent RNA-seq study detected minor up-regulation of Hsp90 by *in hospite Symbiodinium* after 24 h of exposure to 30 °C relative to 23–24 °C, but not after 72 h of exposure to 30 °C (Rosic et al. 2014).

In our study, general down-regulation of thermal tolerance genes was observed on day 9 in both populations at 32 °C (fig. 4*B* and supplementary fig. S2*B* and tables S4 and S5, Supplementary Material online). One *Hsp90* gene, one *Cyp450* gene, and two *DnaJ* genes were up-regulated by the SM population at 32 °C compared with just one *DnaJ* gene up-regulated in the MI population at 32 °C. *Hsp* genes were uniquely found to be down-regulated in the MI population at 32 °C, although the MI population also showed no signs of physiological heat stress throughout the study. Elevated temperature has previously been shown to reduce the expression of *Hsp* genes and *Cyp450* genes in *Symbiodinium* (Rosic et al. 2010; Rosic et al. 2011) as well as the expression of *Hsp* genes and ROS scavenging genes in corals (Rosic et al. 2014; Bay and Palumbi 2015). Down-regulation of some thermal tolerance genes may be attributed to the general down-regulation of > 90% of all DEGs in both populations at 32 °C on day 9. The down-regulated genes in each population were not enriched for GO categories related to thermal tolerance (e.g., unfolded protein binding, the oxidoreductase complex), supporting the notion that down-regulation of thermal tolerance genes may simply reflect a non-targeted, global reduction in transcription to conserve energy at 32 °C.

On day 13, only one glutaredoxin (*Glrx*) gene was up-regulated in the SM population at 32 °C. In contrast, three *Fe-Sod* genes, one cytochrome *c* peroxidase (*Ccpr*) gene, one glutathione peroxidase (*Gpx*) gene, three *Prdx* genes, two thioredoxin (*Txn*) genes, and one *Cyp450* gene were significantly up-regulated in the MI population at 32 °C, highlighting the importance of ROS scavenging genes in type C1 *Symbiodinium* thermal tolerance. Additionally, 11 *Hsp70* genes, four *Hsp90* genes, and eight *DnaJ* genes were up-regulated by the MI population at 32 °C compared with only one *Hsp70* gene, three *Hsp90* genes, and four *DnaJ* genes up-regulated in the SM population at 32 °C (fig. 4*C* and supplementary fig. S2*C* and tables S6 and S7, Supplementary Material online). Up-regulation of *Hsp90* genes by both populations under heat stress is consistent with findings that *Symbiodinium* HSP90 protein abundance increases under heat stress (Ross 2014).

### Linking *Symbiodinium* Transcriptional Heat Stress Responses to Thermal History, Physiological Heat Stress Responses, and Coral Bleaching Susceptibility

The SM and MI *Symbiodinium* populations have been kept in culture for more than 4 years at approximately 27 °C, and their relative thermal tolerances, reported in 2012 (Howells et al. 2012), were confirmed in our current study conducted in 2015. The marked difference in their transcriptional responses to elevated temperature may, therefore, be driven by stable, heritable stress memory due to the different thermal regimes of the SM and MI reefs. Stress memory (or “priming”) is the process in which previous exposure to a particular stress causes epigenetic and/or chromosomal modifications. The modifications allow for a faster and stronger acclimation response to subsequent exposures and can be stably passed on to future generations (Bruce et al. 2007). The warmer MI reef reaches ≥ 32 °C on approximately 12% of summer days, unlike the cooler SM reef where no summer days reach ≥ 32 °C (Howells et al. 2012), suggesting that only the MI population has been primed and/or genetically adapted for efficient acclimation to 32 °C. Successful PCR amplification of a *Fe-Sod* gene from only the genomic DNA of the MI population (supplementary fig. S3*A*, Supplementary Material online) indicates that genetic adaptation is involved in acclimation to 32 °C, but epigenomic and genomic analysis will be necessary to determine whether stress memory also contributes to the transcriptional acclimation response.

**Fig. 5:**
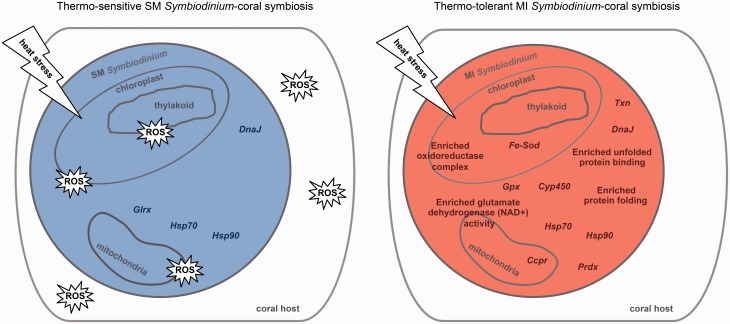
Model of the molecular basis of *Symbiodinium* thermal tolerance and its impacts on *Symbiodinium*-coral symbiosis. Schematics of *Symbiodinium* cells from the SM population and MI population after 13 days at 32 °C hypothesize the impacts of their respective up-regulated thermal tolerance genes (Trinity/edgeR: fold ≥ 4 and FDR ≤ 0.001 relative to 27 °C) and enriched thermal tolerance GO categories (Goseq: FDR < 0.05) on their coral hosts. The main organelles contributing to ROS production are depicted. The shape containing “ROS” represents oxidative damage to the *Symbiodinium* cell or the coral host. Gene abbreviations are as follows: iron superoxide dismutase (*Fe-Sod*), cytochrome *c* peroxidase (*Ccpr*), glutathione peroxidase (*Gpx*), peroxiredoxin (*Prdx*), heat shock protein 70 (*Hsp70*), heat shock protein 90 (*Hsp90*), glutaredoxin (*Glrx*), thioredoxin (*Txn*), cytochrome P450 (*Cyp450*), chaperone protein DnaJ (*DnaJ*).

Acclimation to elevated temperature by the MI population highlights the importance of up-regulating hallmark thermal tolerance genes. Particularly, significant up-regulation of genes for unfolded protein binding, protein folding, and the oxidoreductase complex likely minimizes damage to photosynthetic apparatuses and ROS leakage from cells—both of which were observed in the heat stressed SM population. We hypothesize that the observed transcriptional response by the MI population in culture may also allow the MI population to maintain symbiosis with its coral host at elevated temperature (Howells et al. 2012). Conversely, the observed leakage of ROS out of cells in the SM population due to unsuccessful acclimation to elevated temperature may cause oxidative damage to the coral host, resulting in bleaching as previously seen with corals harboring the SM population when exposed to heat stress (Howells et al. 2012) (fig. 5). Although *Hsp* gene expression has been found to be indistinguishable between *Symbiodinium* in culture and *in hospite* (Rosic et al. 2011), more extensive temporal studies of *in hospite Symbiodinium* gene expression will be necessary to determine the effect of symbiosis on the comprehensive collection of DEGs identified here. Metabolomics should also be utilized to determine if metabolic compensation of *in hospite Symbiodinium* over extended periods of heat stress factors into the breakdown of *Symbiodinium*-coral symbiosis.

In this study, we have detailed gene regulation by a thermo-sensitive type C1 *Symbiodinium* population and a thermo-tolerant type C1 *Symbiodinium* population in response to heat stress that parallels their respective physiological responses to heat stress and previously described bleaching responses *in hospite* (Howells et al. 2012). Furthermore, our study is the first to identify individual genes as well as overarching functional gene groups that influence dinoflagellate thermal tolerance. Our results provide critical insights into the impacts of *Symbiodinium* gene regulation on coral bleaching and present genes (e.g., *Msh4*, *Msh5*, and *Spo11-2*) that could be used to detect heat stress in *Symbiodinium* before potential physiological damage occurs.

## Materials and Methods

### Culture Maintenance and Genotyping

The SM and MI heterogeneous *Symbiodinium* populations (aims-aten-C1-WSY and aims-aten-C1-MI, respectively) were provided by the Symbiont Culture Facility at the Australian Institute of Marine Science and are the same as reported in Howells et al. (2012). Following isolation from *Acropora tenuis*, the *Symbiodinium* populations were initially cultured in filtered seawater supplemented with Daigo IMK (Wako Pure Chemical Industries, Ltd.) and bacterial antibiotics for one month, which minimized the bacterial community to prevent bacterial overgrowth. Cultures were then routinely subcultured in media without antibiotics and monitored regularly by microscopy to ensure no increase in the remaining bacterial community was observed. Complete removal of all bacteria originating from the coral holobiont was not desirable, as optimal growth of dinoflagellate cultures has been shown to require associated bacteria (Alavi et al. 2001; Croft et al. 2005). Dinoflagellate cultures may depend on bacteria to provide necessary components, including but not limited to vitamin B12, in order to thrive (Croft et al. 2005; Ritchie 2012). Unlike the free-living life cycle stage in which *Symbiodinium* naturally live without a coral host (Yamashita and Koike 2013; Granados-Cifuentes et al. 2015), we are not aware of any stage in which *Symbiodinium* naturally live without associated bacteria. Therefore, complete removal of *Symbiodinium*-associated bacteria may have unnatural effects on *Symbiodinium* transcriptomes.

For genotyping of the *Symbiodinium* populations, DNA from cultured cells in exponential growth phase was extracted using the DNeasy Plant Mini Kit (Qiagen). The ITS1 region was amplified with the PCR primers and conditions from van Oppen et al. (2001). The partial 5.8S rDNA, ITS2, and partial 28S rDNA region was amplified with the PCR primers and conditions from Stat et al. (2009). Purified PCR products were sequenced by the Australian Genome Research Facility. The ITS1 and ITS2 sequences of each population were reconfirmed to be type C1 and to be identical between the SM and MI populations, as previously reported by Howells et al. (2012). In future studies, alternative molecular markers such as the non-coding region from the psbA minicircle could be assessed through next generation sequencing to investigate finer-scale evolutionarily divergence that may exist between the two C1 populations as well as within each C1 population (LaJeunesse and Thornhill 2011). Increased genetic resolution may provide valuable insight into the different transcriptional responses to heat stress observed in our study.

### Experimental Setup

Each population (∼1 × 10^6^ cells/ml, 50 ml total volume) was added to eight replicate culture flasks (*n* = 4 for each temperature treatment, supplementary table S1, Supplementary Material online). Two flasks per population were randomly assigned to each of four experimental incubators and acclimated at 27 °C. Light was provided to cells at an intensity of 30 µmol quanta m^−2^ s^−1^ (Crompton 36W cool white fluorescent tubes, 4000 K) with a 12:12 h light:dark cycle. After 10 days of acclimation, fresh media was supplied to the cultures. Following an additional four days of acclimation (two weeks of acclimation total), two incubators were ramped on day 0 at 0.5 °C/h to 32 °C for the heat stress temperature treatment, whereas two incubators remained at 27 °C for the control temperature treatment. Temperature and light intensity in the incubators were monitored with HOBO data loggers (Onset Computer Corporation). Cultures remained in exponential growth phase, determined by the average of three replicate haemocytometer counts for each sample recorded throughout the experiment (supplementary fig. S1*D*, Supplementary Material online).

### Photosynthesis Measurements

A Mini-PAM fluorometer (Walz, Germany) was used to measure effective quantum yield (supplementary fig. S1*A*, Supplementary Material online) and rapid light curves (RLCs) (fig. 1*A* and *B* and supplementary fig. S1*B*, Supplementary Material online). RLCs are ideal for providing quick snapshots of *Symbiodinium* responses to a range of irradiances with results that are reasonably comparable to steady-state light curves (Suggett et al. 2015). With RLCs, < 90 s of exposure to high irradiances is applied per sample, which was short enough to avoid significant long-term damage that could greatly affect the physiology and gene expression of the *Symbiodinium* and allowed for the concurrent analysis of all 16 samples within an equivalent period of the light cycle on each measurement day.

A RLC protocol adapted from Ralph et al. (2002) was used in our study. After 7 h of light exposure, the fiber–optic cable of the Mini-PAM fluorometer was held against the bottom of each culture flask where the *Symbiodinium* cells had settled. *Symbiodinium* were exposed to nine steps of increasing actinic light (0–1,775 μmol m^−2^ s^−1^ PAR) for 10 s each, separated by a saturating pulse (0.8 s, > 4,000 μmol m^−2^ s^−1^ PAR). The light responses of each population at each temperature were determined by fitting the RLCs to the model by Platt et al. (1980). The variables rETRm, α, and *E*_k_ (fig. 1*A* and *B* and supplementary fig. S1*B*, Supplementary Material online) were calculated using SigmaPlot as per Hill et al. (2004).

### ROS Measurements

Cultures were gently agitated to evenly distribute cells in the media, and aliquots (300 µl per sample) were centrifuged at 3,000*g* × 5 min. Media (for measuring ROS leakage) were collected without disturbing the cell pellet and incubated with CellROX® Orange reagent (5 µM, Thermo Fisher Scientific) for oxidative stress detection in a 96-well black clear bottom plate (Costar) for 2 h at 27 °C in the dark. CellROX® reagent is irreversibly converted to a fluorescent state in the presence of ROS without requiring the activity of intracellular esterases, making it an appropriate dye for measuring general ROS content in media. Fluorescence intensity of the CellROX® reagent was measured at excitation 540 nm and emission 565 nm with an EnSpire® Multimode Plate Reader (PerkinElmer). The use of CellROX® reagent with *Symbiodinium* culture media was validated through CellROX® reagent signal quenching from addition of antioxidant chemicals (supplementary fig. S4, Supplementary Material online).

### Culture Viability Measurements

Culture viability was measured with SYTOX® Green nucleic acid stain (Life Technologies), which is unable to penetrate live *Symbiodinium* cells. Cultures were gently agitated to evenly distribute cells in the media, and aliquots (50 µl per sample) were incubated with SYTOX® Green nucleic acid stain (1 μM) in the dark for 15 min. An Olympus fv1000 confocal microscope with a 488 nm argon-ion laser was used to quantify the proportion of live cells in each sample based on counts of stained and unstained cells averaged across three separate fields of view (supplementary fig. S1*C*, Supplementary Material online).

### Statistical Analysis of Physiological Measurements

The PRIMER software with the PERMANOVA+ package was used to determine significant differences (*P* < 0.05) between temperature treatments for each physiological measurement using PERMANOVA with two replicate incubators as a nested factor within each level of the factor temperature (27 °C and 32 °C) and two flasks of each population in each incubator for each temperature. Where the effect of incubators was not significant (*P* > 0.2), the incubator factor was pooled, and each temperature treatment within each population (*n* = 4) was compared using a one-way PERMANOVA.

### Preparation and Sequencing of RNA Samples

Precisely after 6 h of light exposure, cultures were gently agitated to evenly distribute cells in the media. Aliquots containing 2–4 × 10^6^ cells per sample were immediately snap frozen in liquid nitrogen within 10 s of removal from the experimental incubators. Instant snap freezing of *Symbiodinium* cells that were still in media (rather than the standard method of pelleting by centrifugation for 5–10 min, removing media, and then snap freezing, Rosic and Hoegh-Guldberg 2010; Baumgarten et al. 2013; Krueger et al. 2015) caused no sign of cell lysis or loss of RNA integrity (supplementary fig. S5, Supplementary Material online). We developed this method to ensure that the effects of experimental temperature treatments on gene expression remained unaltered during sample preservation because gene expression can be affected by centrifugation and extended handling (Baldi and Hatfield 2002). Our method is the only one of which we are aware to immediately preserve *Symbiodinium* RNA since the compatibility of RNAlater (Thermo Fisher Scientific) with *Symbiodinium* has not yet been validated. Samples were stored at −80 °C until completion of the heat stress experiment and were processed together on the same day to prevent batch effect.

Snap frozen cells were thawed at room temperature and pelleted at 4 °C (3,000*g* × 5 min). Media were removed, and pellets were lysed in buffer RLT (RNeasy Plant Mini Kit, Qiagen) containing β-mercaptoethanol by bead beating with 0.3 g of 710–1,180 µm acid-washed glass beads (Sigma) using a TissueLyser II (Qiagen) for 90 s at 30 Hz. RNA was then extracted and purified using the RNeasy Plant Mini Kit (Qiagen) with an added on-column DNase I treatment (Qiagen). Total RNA (150–500 ng) of each sample was sent to the Australian Genome Research Facility for confirmation of high quality RNA using an Agilent 2100 bioanalyzer, polyA-purification, Illumina TruSeq stranded library preparation, and sequencing with an Illumina HiSeq2500 (single end 100 bp, ∼10^7^ reads per sample, supplementary table S2, Supplementary Material online).

### Transcriptome Assembly and Differential Gene Expression Analysis

Illumina Truseq (TruSeq3-SE) adapters were removed from RNA sequence reads using Trimmomatic (Bolger et al. 2014). Prinseq (Schmieder and Edwards 2011) was then used to remove poly-A tails (min tail: 6-A) and to filter out short (min length: 60 bp), low quality (min mean quality score: 20, base window: 1, base step: 1), and low complexity sequences (dust method threshold: 7). The sequence reads for the 24 samples per population (four replicates, two temperature treatments, three time points) that remained after quality filtering were combined for *de novo* assembly of the SM population transcriptome and MI population transcriptome using Trinity (Grabherr et al. 2011; Haas et al. 2013) (version: 2.0.6). Minimum transcript length for *de novo* assembly was set to 150 bp. To focus on transcripts with higher coverage, only transcripts ≥ 250 bp were retained for analysis, as in Baumgarten et al. (2013). Redundant transcripts (99% sequence similarity over 99% of the shorter transcript) in each *de novo* assembly were collapsed into the longest representative transcript using cd-hit-est (Huang et al. 2010) (supplementary table S2, Supplementary Material online). Completeness of the SM, MI, and other publicly accessible, published *Symbiodnium* transcriptomes (Bayer et al. 2012; Ladner et al. 2012; Baumgarten et al. 2013; Rosic et al. 2015; Xiang et al. 2015; Parkinson et al. 2016) was assessed using BUSCO with the set of 429 conserved eukaryotic orthologs that have been found to be present in > 90% of surveyed eukaryotic species (though the surveyed species currently lack protist representatives leading BUSCO to be biased towards lower metrics for protists than would otherwise be expected) (Simão et al. 2015) (supplementary table S3, Supplementary Material online). Non-redundant (nr) genes (transcript clusters determined by Trinity based on shared sequence content) were then analyzed for differential expression (fold ≥ 4 and FDR ≤ 0.001 between temperature treatments) according to the standard Trinity pipeline (Haas et al. 2013) (https://github.com/trinityrnaseq/trinityrnaseq/wiki, last accessed June, 2016) using RSEM (Li and Dewey 2011) and edgeR (Robinson et al. 2010). Additionally, the BCV of expression counts for all genes across replicates at each time point was separately calculated in edgeR according to Chen et al. (2014).

### Annotation and GO Analysis

Transcriptomes were functionally annotated with Trinotate (http://trinotate.github.io/, last accessed June, 2016), using the SwissProt and UniRef90/TrEMBL databases (NCBI BLAST+, e-value ≤ 10^−5^) and the Pfam-A database (HMMR, domain noise cutoff). Top hits from SwissProt were used to annotate transcripts. If a hit was not generated against SwissProt, then the top hit from UniRef90/TrEMBL determined annotation. In the absence of a UniRef90/TrEMBL hit, Pfam-A annotation was used. GOseq (Young et al. 2010), which corrects for transcript length bias, was used as detailed with Trinity (https://github.com/trinityrnaseq/trinityrnaseq/wiki/Running_GOSeq, last accessed June, 2016) for GO analysis (FDR < 0.05, ancestral terms included) of DEGs (fold ≥ 4 and FDR ≤ 0.001 between temperature treatments). SwissProt was used to assign GO categories. In the absence of a SwissProt assignment, GO categories provided by Pfam-A were used.

In the SM and MI populations, 33% and 34% of genes received a hit from SwissProt, 46% and 49% of genes received a hit from UniRef90/TrEMBL, and 34% and 36% of genes received a hit from Pfam-A; respectively. In total, 50% of genes in the SM population and 52% of genes in the MI population received annotation from at least one database, and 35% of genes in the SM population and 36% of genes in the MI population were annotated with GO categories—similar to what has been previously reported for annotation of other *Symbiodinium* transcriptomes (Baumgarten et al. 2013; Rosic et al. 2015; Parkinson et al. 2016). Raw sequence reads, assembled transcriptomes, gene read count matrices, and transcript annotation results are available on NCBI GEO (GEO: GSE72763).

### Isolation of a *Fe-Sod* Gene

The SM and MI populations were cultured for one week in sterile media with 300 μg/ml of ampicillin, followed by one week in sterile media with 300 μg/ml kanamycin, and finally for one week in sterile media with 100 μg/ml spectinomycin. Afterwards, *Symbiodinium* cells were pelleted (3,000*g* × 5 min) and washed three times in sterile media. Genomic DNA was extracted with a PureLink® Genomic DNA Mini Kit (Thermo Fisher Scientific). To confirm that the genomic DNA was of high quality and amplifiable, ITS2 PCR primers (Stat et al. 2011) were successfully used to amplify the ITS2 region from 25 ng of SM or MI genomic DNA. Primers for amplification of a full-length *Symbiodinium Fe-Sod* gene were based on TR20255|c0_g1 (open reading frame: 674-78[-]) from the MI population (forward: 5′ ATG GCC TTC TCC ATC CCA CCG 3′; reverse: 5′ TCA CAG GTT GGA CTC GGC GAA C 3′) and used for PCR reactions containing 125 ng of SM or MI genomic DNA (supplementary fig. S3*A*, Supplementary Material online). The purified *Fe-Sod* PCR product that was amplified from the MI genomic DNA was sequenced by the Australian Genome Research Facility and confirmed to match TR20255|c0_g1 (open reading frame: 674-78[-]). The sequence of TR20255|c0_g1 (open reading frame: 674-78[-]) was aligned to the sequences of *Symbiodinium Fe-Sod* genes identified by Krueger et al. (2015) using ClustalW (Thompson et al. 2002). Alignments were visualized with UCSF Chimera (Pettersen et al. 2004) (supplementary fig. S3*B*, Supplementary Material online).

## Supplementary Material

Supplementary material figures S1–S5, tables S1–S7 and dataset S1 is available at *Molecular Biology and Evolution* online (http://www.mbe.oxfordjournals.org/).

Supplementary Data
