# Supplementary material for: Sex, Scavengers, and Chaperones: Transcriptome Secrets of Divergent Symbiodinium Thermal Tolerances
Source: Mol Biol Evol. 2016 Jun 14;33(9):2201–15. doi: 10.1093/molbev/msw119 (PMC4989115; doi:10.1093/molbev/msw119)
Supplement: Supplementary Data [file supp_33_9_2201__index.html]

Sex, Scavengers, and Chaperones: Transcriptome Secrets of Divergent Symbiodinium Thermal Tolerances — Sex, Scavengers, and Chaperones: Transcriptome Secrets of Divergent Symbiodinium Thermal Tolerances — Supplementary Data 

# Sex, Scavengers, and Chaperones: Transcriptome Secrets of Divergent *Symbiodinium* Thermal Tolerances

## Supplementary Data

files

- Supplementary Data - xlsx file
- Supplementary Data - pdf file
