# Supplementary material for: Sex, Scavengers, and Chaperones: Transcriptome Secrets of Divergent Symbiodinium Thermal Tolerances
Source: Mol Biol Evol. 2016 Jun 14;33(9):2201–15. doi: 10.1093/molbev/msw119 (PMC4989115; doi:10.1093/molbev/msw119)
Supplement: Supplementary Data [file supp_msw119_Levin_2016_SupplementaryMaterial.pdf]

SUPPLEMENTARY TABLES

Table S1.
Experimental design and sampling time points for transcriptomics.

Population	Temperature	Incubator	Day -1	Day 9	Day 13
SM	27°C	A	2 samples	2 samples	2 samples
SM	27°C	B	2 samples	2 samples	2 samples
SM	32°C	C	2 samples	2 samples	2 samples
SM	32°C	D	2 samples	2 samples	2 samples
MI	27°C	A	2 samples	2 samples	2 samples
MI	27°C	B	2 samples	2 samples	2 samples
MI	32°C	C	2 samples	2 samples	2 samples
MI	32°C	D	2 samples	2 samples	2 samples

Table S2.
De novo assemblies of *Symbiodinium* population transcriptomes.

Population	Raw reads	Filtered reads[a]	Assembled transcripts	Nr assembled transcripts[b] (≥ 250 bp)	Nr genes[c] (≥ 250 bp)	N50[d] (≥ 250 bp)	Average nr transcript length (≥ 250 bp)	Read alignment[e]
SM	238,256,872	236,965,564	205,494	131,066	106,097	1,253	858.1	88.0%
MI	235,479,270	234,027,212	183,989	116,479	93,377	1,353	911.4	89.6%

[a] Number of reads retained after quality filtering of raw sequence reads
[b] Number of non-redundant (nr) transcripts after removing transcripts < 250 bp and collapsing highly similar transcripts
[c] Transcript (putative isoform) clusters based on shared sequence content
[d] Weighted median statistic of transcript length (50% of the nr transcripts ≥ the N50 value)
[e] Proportion of filtered reads that mapped back to the nr transcripts

Table S3.

Completeness of publicly accessible, published *Symbiodinium* transcriptomes based on the presence of 429 eukaryotic benchmarking universal single-copy orthologs (BUSCOs) from OrthoDB (C: complete [D: duplicated], F: fragmented, M: missing). Asterisks mark transcriptomes that were generated in this study.

***Symbiodinium* type**	**Reference**	**C[D]**	**F**	**M**
A1 (*S. microadriaticum*)	Baumgarten et al. (2013)	55%[38%]	13%	32%
A2	Rosic et al. (2015)	16%[3%]	22%	62%
A	Bayer et al. (2012)	47%[8%]	14%	39%
B1 (*S. minutum*)	Bayer et al. (2012)	30%[8%]	19%	51%
B1 (*S. minutum*)	Parkinson et al. (2016)	65%[13%]	6%	29%
B1 (*S. pseudominutum*)	Parkinson et al. (2016)	59%[12%]	8%	33%
B19 (*S. psygmophilum*)	Parkinson et al. (2016)	66%[13%]	5%	29%
B19 (*S. aenigmaticum*)	Parkinson et al. (2016)	53%[10%]	11%	36%
B2	Rosic et al. (2015)	14%[6%]	25%	61%
B	Xiang et al. (2015)	68%[16%]	4%	28%
C1	Rosic et al. (2015)	26%[5%]	24%	50%
C1 (SM population)*	Levin et al. (2016)	71%[32%]	7%	22%
C1 (MI population)*	Levin et al. (2016)	72%[30%]	5%	23%
C3k	Ladner et al. (2012)	16%[3%]	23%	61%
D1	Rosic et al. (2015)	26%[5%]	29%	45%
D2	Ladner et al. (2012)	26%[5%]	22%	52%

Table S4.

Up-regulated (+) and down-regulated (-) meiosis, ROS scavenging, and molecular chaperone genes in the SM population after 9 days at 32°C (fold ≥ 4 and FDR ≤ 0.001 relative to 27°C).

**Gene ID**	**Gene annotation**	**$\log_2$(fold)**	**Counts per million reads**	**FDR**
TR26080|c0_g1	MutS protein homolog 4	+2.52	6.17	1.95E-08
TR15578|c0_g1	MutS protein homolog 5	+2.24	14.79	8.26E-14
TR41849|c0_g2	Meiotic recombination protein Spo11-2	+2.32	1.77	2.06E-05
TR72293|c1_g1	Heat shock protein 83	+2.75	2.36	6.13E-09
TR47492|c0_g1	Cytochrome P450	+4.06	0.67	5.65E-05
TR63594|c0_g1	DnaJ homolog subfamily B member 6-B	+2.73	1.44	4.21E-06
TR73294|c0_g1	DnaJ homolog subfamily C member 7	+3.08	2.51	2.88E-10
TR90535|c0_g1	Superoxide dismutase [Mn]	-2.28	9.51	7.25E-05
TR107728|c0_g1	Catalase-peroxidase	-4.09	0.58	3.33E-04
TR58725|c0_g2	Probable L-ascorbate peroxidase 4	-2.15	11.12	9.54E-04
TR67510|c0_g1	Glutathione peroxidase 2	-2.42	1.11	1.52E-04

TR48775|c0_g1	Peroxiredoxin-6	-3.55	3.23	2.22E-10
TR49236|c0_g1	Peroxiredoxin-2B	-2.86	1.05	9.99E-05
TR65128|c0_g1	Peroxiredoxin-2D	-2.56	2.63	1.59E-04
TR92045|c0_g1	Peroxiredoxin TSA1	-2.69	0.87	2.15E-04
TR77174|c0_g1	Alternative oxidase	-2.26	4.41	4.24E-04
TR60729|c0_g2	Glutaredoxin	-2.45	1.29	6.99E-05
TR106479|c0_g1	Monothiol glutaredoxin-S15	-4.12	1.07	8.36E-08
TR22361|c0_g1	Thioredoxin	-2.50	1.52	2.62E-05
TR13040|c0_g1	Thioredoxin	-2.06	1.79	2.44E-05
TR2965|c0_g1	Thioredoxin	-5.06	0.46	4.61E-04
TR127388|c0_g1	Thioredoxin-1	-2.63	0.75	4.04E-04
TR48398|c0_g1	Thioredoxin domain-containing protein	-3.12	1.18	3.17E-06
TR46153|c0_g1	Thioredoxin domain-containing protein	-2.52	1.99	1.05E-05
TR49313|c0_g2	Cytochrome P450	-3.01	4.00	1.11E-06
TR9469|c0_g1	DnaJ homolog subfamily A member 4	-2.47	2.73	2.78E-07

Table S5.

Up-regulated (+) and down-regulated (-) meiosis, ROS scavenging, and molecular chaperone genes in the MI population after 9 days at 32°C (fold ≥ 4 and FDR ≤ 0.001 relative to 27°C).

Gene ID	Gene annotation	$\log_2$(fold)	Counts per million reads	FDR
TR3359|c0_g1	MutS protein homolog 4	+2.65	4.20	2.50E-14
TR34615|c0_g1	MutS protein homolog 5	+2.12	10.83	2.23E-19
TR47116|c0_g1	Meiotic recombination protein Spo11-2	+2.21	4.51	6.19E-11
TR57555|c0_g1	Molecular chaperone DnaJ	+2.12	1.90	2.20E-07
TR49599|c0_g2	Superoxide dismutase [Cu-Zn]	-2.40	2.13	5.69E-11
TR35071|c0_g1	Superoxide dismutase [Fe]	-2.74	0.70	7.62E-04
TR47713|c0_g1	Cytochrome c peroxidase, mitochondrial	-2.47	2.11	1.36E-06
TR18639|c0_g1	Peroxiredoxin-2	-2.25	1.48	2.39E-07
TR91822|c0_g1	Peroxiredoxin-2C	-3.89	0.78	9.12E-06
TR99969|c0_g1	Peroxiredoxin-2F, mitochondrial	-2.47	0.82	1.54E-04
TR61543|c0_g1	Thioredoxin peroxidase	-2.55	1.24	1.26E-06
TR121144|c0_g1	Heat shock 70 kDa protein	-2.08	4.56	8.52E-07
TR41580|c0_g1	Heat shock 70-related protein 1	-2.04	1.05	4.39E-04
TR44032|c0_g1	Heat shock protein SSA3	-2.48	1.38	3.79E-05
TR50028|c0_g1	Heat shock 70 kDa protein, mitochondrial	-2.19	1.28	4.48E-05
TR58449|c0_g1	Heat shock 70 kDa protein 4	-2.53	1.32	2.58E-05
TR62486|c1_g1	Heat shock 70 kDa protein 4	-2.51	13.04	1.71E-09
TR71496|c0_g1	Heat shock cognate 70 kDa protein	-2.49	1.11	1.49E-05

TR63145|c0_g1	Heat shock protein hsp88	-2.25	1.15	1.67E-04
TR56344|c0_g1	Heat shock-like 85 kDa protein	-2.14	3.08	1.88E-04
TR59291|c0_g1	Heat shock-like 85 kDa protein	-2.17	9.03	2.42E-05
TR64058|c0_g1	Heat shock protein 81-3	-2.17	3.61	6.56E-06
TR11461|c0_g1	Thioredoxin-1	-3.04	1.19	3.06E-07
TR9775|c0_g1	Thioredoxin domain-containing protein 12	-3.33	0.76	1.28E-05
TR112961|c0_g1	Thioredoxin domain-containing protein 5	-2.07	5.77	2.74E-06
TR25578|c0_g2	Thioredoxin-like protein 1	-2.64	0.68	6.37E-04
TR82304|c0_g1	Cytochrome P450 4V2	-2.99	0.66	5.16E-04
TR3328|c0_g2	Cytochrome P450 4V2	-3.85	1.64	1.27E-09
TR63302|c0_g1	Sterol 14-alpha demethylase	-2.25	0.99	8.92E-05
TR16011|c0_g2	DnaJ homolog subfamily B member 6-A	-4.05	0.59	9.15E-05
TR58329|c0_g1	DnaJ homolog subfamily A member 1	-2.40	1.51	6.79E-06
TR52403|c0_g1	DnaJ homolog subfamily A member 2	-2.58	2.14	1.31E-09
TR60716|c0_g1	DnaJ homolog subfamily A member 4	-2.92	0.76	9.61E-05

Table S6.

Up-regulated (+) and down-regulated (-) meiosis, ROS scavenging, and molecular chaperone genes in the SM population after 13 days at 32°C (fold ≥ 4 and FDR ≤ 0.001 relative to 27°C).

**Gene ID**	**Gene annotation**	**$\log_2$(fold)**	**Counts per million reads**	**FDR**
TR26080|c0_g1	MutS protein homolog 4	+3.69	8.22	6.95E-44
TR15578|c0_g1	MutS protein homolog 5	+2.18	14.06	1.96E-28
TR41849|c0_g1	Meiotic recombination protein Spo11-2	+4.27	0.73	8.85E-06
TR41849|c0_g2	Meiotic recombination protein Spo11-2	+3.19	2.69	3.47E-16
TR72002|c0_g4	Heat shock protein 105 kDa	+2.04	5.61	9.77E-11
TR65031|c0_g1	Endoplasmin homolog	+2.00	2.37	2.60E-07
TR58135|c0_g1	Heat shock protein 90-1	+4.93	0.47	4.82E-04
TR72293|c1_g1	Heat shock protein 83	+2.67	2.29	1.44E-08
TR56879|c0_g1	Glutaredoxin-C2	+2.21	1.03	3.30E-04
TR14920|c0_g1	Chaperone protein DnaJ	+2.60	0.88	9.47E-05
TR63594|c0_g1	DnaJ homolog subfamily B member 6-B	+4.05	1.27	4.09E-08
TR73294|c0_g1	DnaJ homolog subfamily C member 7	+2.96	2.22	5.56E-09
TR55386|c0_g2	DnaJ protein homolog	+2.42	1.03	5.95E-05
TR90535|c0_g1	Superoxide dismutase [Mn]	-2.24	3.18	5.61E-04
TR61288|c0_g1	Probable phospholipid hydroperoxide glutathione peroxidase 6	-2.71	1.03	4.24E-04
TR94914|c0_g1	Glutaredoxin-C4	-4.20	0.59	2.90E-04
TR8663|c0_g1	Peroxiredoxin Q, chloroplastic	-2.48	0.77	3.63E-04

TR73087|c0_g1	Heat shock 70 kDa protein 14	-2.05	17.71	1.30E-04
TR1229|c0_g1	Heat shock 70 kDa protein	-2.27	31.00	1.66E-05
TR92001|c0_g1	Heat shock 70 kDa protein	-2.73	20.20	6.80E-06
TR126276|c0_g1	Heat shock protein SSA3	-3.20	24.45	9.35E-10
TR60729|c0_g2	Glutaredoxin	-2.40	1.00	3.55E-05
TR33105|c0_g3	Glutaredoxin-1	-2.08	12.46	5.78E-27
TR25363|c0_g1	Glutaredoxin-1	-5.44	0.52	3.73E-04
TR106479|c0_g1	Monothiol glutaredoxin-S15, mitochondrial	-3.99	0.55	1.73E-04
TR22361|c0_g1	Thioredoxin	-3.11	0.65	1.06E-04
TR27587|c0_g2	Thioredoxin	-3.32	1.69	7.15E-11
TR13040|c0_g1	Thioredoxin	-3.41	1.03	2.76E-05
TR49951|c0_g1	Cytochrome P450 704C1	-3.33	0.71	8.99E-05
TR73168|c0_g1	Probable cytochrome P450 49a1	-2.92	0.94	1.57E-06
TR70469|c0_g1	Cytochrome P450 3A13	-2.08	1.13	1.72E-04
TR73331|c0_g1	Cytochrome P450 3A19	-2.82	2.25	5.98E-12
TR49313|c0_g2	Cytochrome P450	-2.34	1.18	5.08E-04
TR74009|c0_g1	DnaJ homolog subfamily B member 14	-3.15	2.06	7.91E-14
TR96409|c0_g1	Chaperone protein DnaJ	-2.42	1.00	3.85E-04
TR56702|c1_g1	DnaJ homolog subfamily A member 1	-2.72	1.52	5.30E-05
TR56318|c0_g2	DnaJ homolog subfamily B member 5	-2.91	6.68	7.58E-11

Table S7.

Up-regulated (+) and down-regulated (-) meiosis, ROS scavenging, and molecular chaperone genes in the MI population after 13 days at 32°C (fold ≥ 4 and FDR ≤ 0.001 relative to 27°C).

**Gene ID**	**Gene annotation**	**log_2(fold)**	**Counts per million reads**	**FDR**
TR3359|c0_g1	MutS protein homolog 4	+2.44	4.26	4.43E-14
TR47116|c0_g1	Meiotic recombination protein Spo11-2	+2.07	5.02	1.95E-09
TR60336|c0_g1	Superoxide dismutase [Fe]	+2.03	1.37	2.70E-05
TR20255|c0_g1	Superoxide dismutase [Fe]	+2.35	3.02	1.14E-08
TR53519|c0_g1	Superoxide dismutase [Fe]	+3.15	0.76	7.18E-05
TR46907|c0_g1	Cytochrome c peroxidase, mitochondrial	+2.24	1.60	3.15E-06
TR55391|c0_g1	Probable phospholipid hydroperoxide glutathione peroxidase	+4.01	0.63	6.15E-05
TR31467|c0_g1	Peroxiredoxin-2	+3.12	1.05	2.08E-06
TR48822|c0_g1	Peroxiredoxin	+2.20	1.40	4.56E-04
TR61543|c0_g1	Thioredoxin peroxidase	+2.44	6.55	8.22E-15
TR40730|c0_g1	Heat shock cognate 70 kDa protein 1	+5.47	0.58	5.81E-05

TR41580|c0_g1	Heat shock 70-related protein 1	+2.59	4.33	3.24E-08
TR43532|c0_g1	Heat shock cognate 70 kDa protein 3	+3.75	0.56	7.31E-04
TR44032|c0_g1	Heat shock protein SSA3	+2.33	4.74	1.33E-12
TR50028|c0_g1	Heat shock 70 kDa protein, mitochondrial	+2.55	6.64	3.41E-20
TR59506|c0_g1	Hsp70 protein	+2.69	1.68	1.09E-06
TR61972|c0_g1	Heat shock cognate 71 kDa protein	+2.52	15.11	1.32E-22
TR62486|c0_g3	Heat shock 70 kDa protein, mitochondrial	+2.04	7.48	2.49E-12
TR62486|c1_g1	Heat shock 70 kDa protein 4	+2.64	71.80	1.39E-16
TR63145|c0_g1	Heat shock protein hsp88	+2.39	4.43	1.38E-14
TR89622|c0_g1	Heat shock cognate 70 kDa protein 3	+3.58	0.73	3.98E-04
TR56344|c0_g1	Heat shock-like 85 kDa protein	+2.32	11.17	3.36E-15
TR59291|c0_g1	Heat shock-like 85 kDa protein	+2.24	32.54	6.03E-16
TR64058|c0_g1	Heat shock protein 81-3	+2.51	15.46	1.11E-25
TR110286|c0_g1	TNF receptor-associated protein 1 homolog, mitochondrial	+2.70	2.38	1.57E-09
TR59710|c0_g1	Thioredoxin-1	+2.29	0.92	3.76E-04
TR51677|c0_g1	Thioredoxin-1	+2.19	1.22	9.42E-06
TR62574|c0_g1	Probable cytochrome P450 12c1, mitochondrial	+2.18	1.56	3.90E-06
TR50785|c0_g1	Chaperone protein DnaJ	+2.73	1.24	1.24E-06
TR16011|c0_g2	DnaJ homolog subfamily B member 6-A	+2.02	1.21	2.38E-04
TR80199|c0_g1	DnaJ homolog subfamily A member 2	+2.57	1.68	1.11E-06
TR61668|c1_g2	DnaJ homolog subfamily A member 2	+2.64	1.97	5.65E-07
TR51427|c0_g1	DnaJ homolog subfamily A member 4	+2.66	1.19	9.90E-05
TR60716|c0_g1	DnaJ homolog subfamily A member 4	+2.25	1.79	3.88E-06
TR40249|c0_g1	DnaJ homolog subfamily C member 3	+2.92	1.89	6.65E-06
TR10977|c0_g1	DnaJ homolog subfamily C member 7 homolog	+2.85	0.93	1.17E-05
TR47513|c0_g1	DnaJ protein homolog 2	+2.89	0.95	7.80E-05
TR49599|c0_g2	Superoxide dismutase [Cu-Zn]	-4.08	0.90	4.39E-04
TR47713|c0_g1	Cytochrome c peroxidase, mitochondrial	-2.89	1.05	1.01E-05
TR899|c0_g1	Cytochrome c peroxidase, mitochondrial	-3.28	0.79	2.64E-04
TR54503|c0_g1	Glutathione peroxidase 1	-4.37	0.72	1.92E-05
TR18639|c0_g1	Peroxiredoxin-2	-3.96	0.86	4.00E-04
TR26880|c0_g1	Heat shock protein 88	-3.22	0.93	2.84E-04
TR4879|c0_g1	Heat shock protein 82	-2.23	2.08	2.88E-05
TR56256|c0_g1	Heat shock cognate 90 kDa protein	-2.90	2.88	3.05E-05
TR112961|c0_g1	Thioredoxin domain-containing protein 5	-2.38	2.91	6.38E-05
TR98120|c0_g1	DnaJ protein P58IPK homolog	-4.22	0.67	2.34E-05
TR52403|c0_g1	DnaJ homolog subfamily A member 2	-2.96	1.09	2.34E-05

SUPPLEMENTARY FIGURES

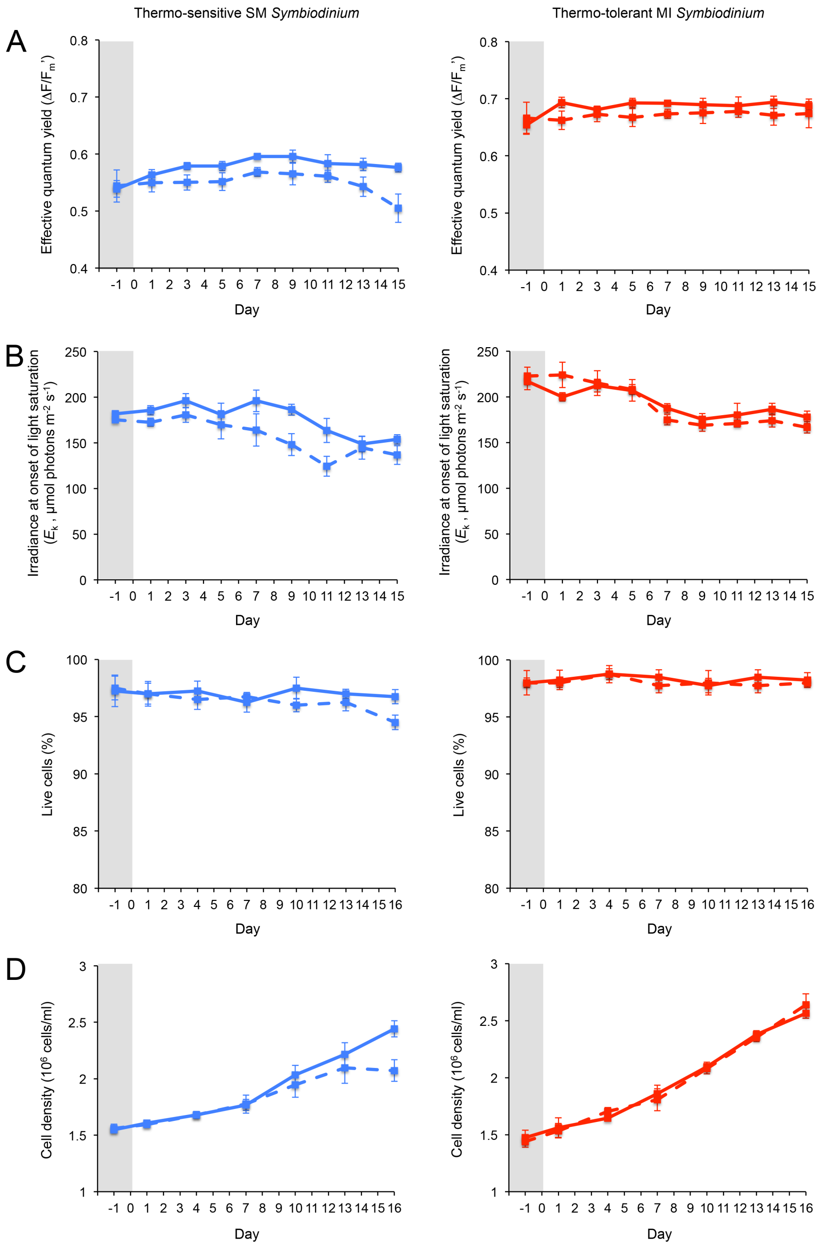

Figure S1.

Additional physiological measurements of *Symbiodinium* exposed to heat stress.

Intact lines represent the 27°C temperature treatment, and dashed lines represent the 32°C temperature treatment. Before heating, all samples were kept at 27°C (values in the grey regions). (A) $\Delta F/F_m'$ (mean ± s.e.m., n = 4). (B) E_k (mean ± s.e.m., n = 4). (C) Culture viability (mean ± s.e.m., n = 4). (D) Cell density (mean ± s.e.m., n = 4). No statistically significant (PERMANOVA) differences were detected between temperature treatments at $p < 0.05$.

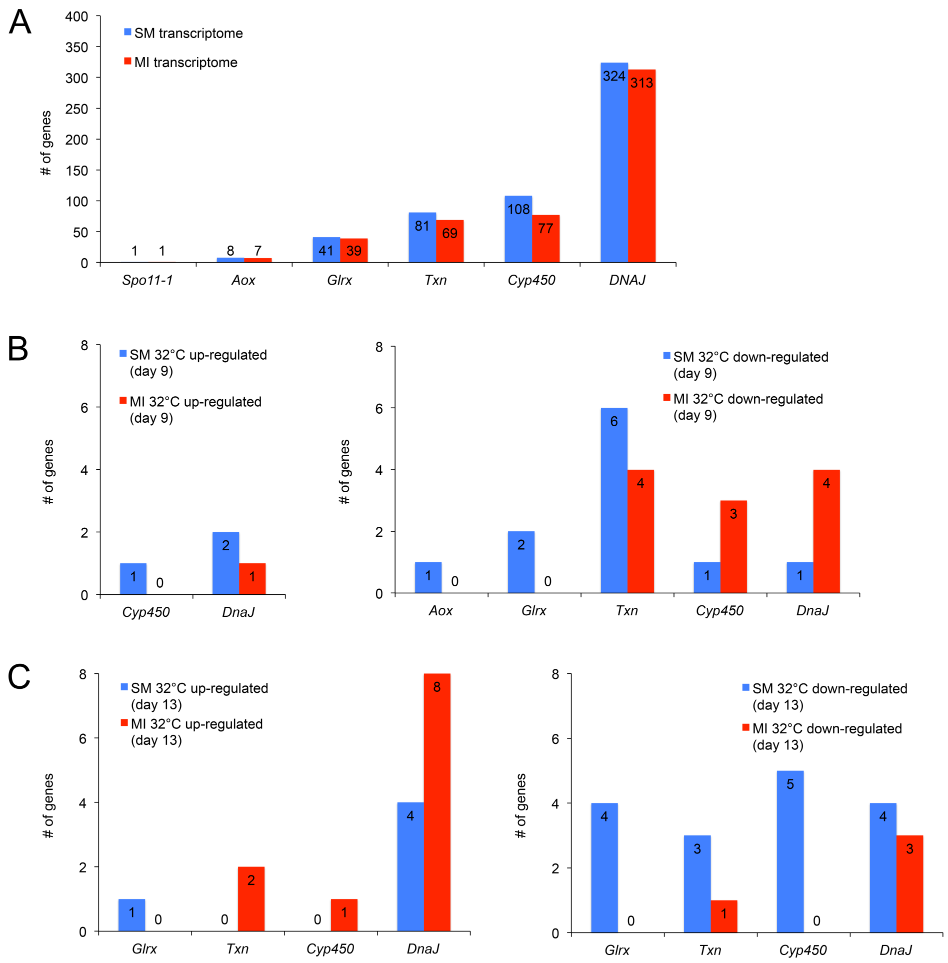

Figure S2.

Regulation of additional meiosis, ROS scavenging, and molecular chaperone genes.

(A) The number of genes for gene types involved in sexual reproduction or thermal tolerance in the SM and MI transcriptomes. The number of DEGs from each gene type at 32°C (Trinity/edgeR: fold ≥ 4 and FDR ≤ 0.001 relative to 27°C) are shown for each population on (B) day 9 and (C) day 13. Gene types that had no DEGs in either population are excluded from (B) and (C). Gene abbreviations are as follows: meiotic recombination protein Spo11-1 (*Spo11-1*), alternative oxidase (*Aox*), glutaredoxin (*Glrx*), thioredoxin (*Txn*), cytochrome P450 (*Cyp450*), chaperone protein DnaJ (*DnaJ*). DEG annotation and differential expression details are provided in Tables S4-7.

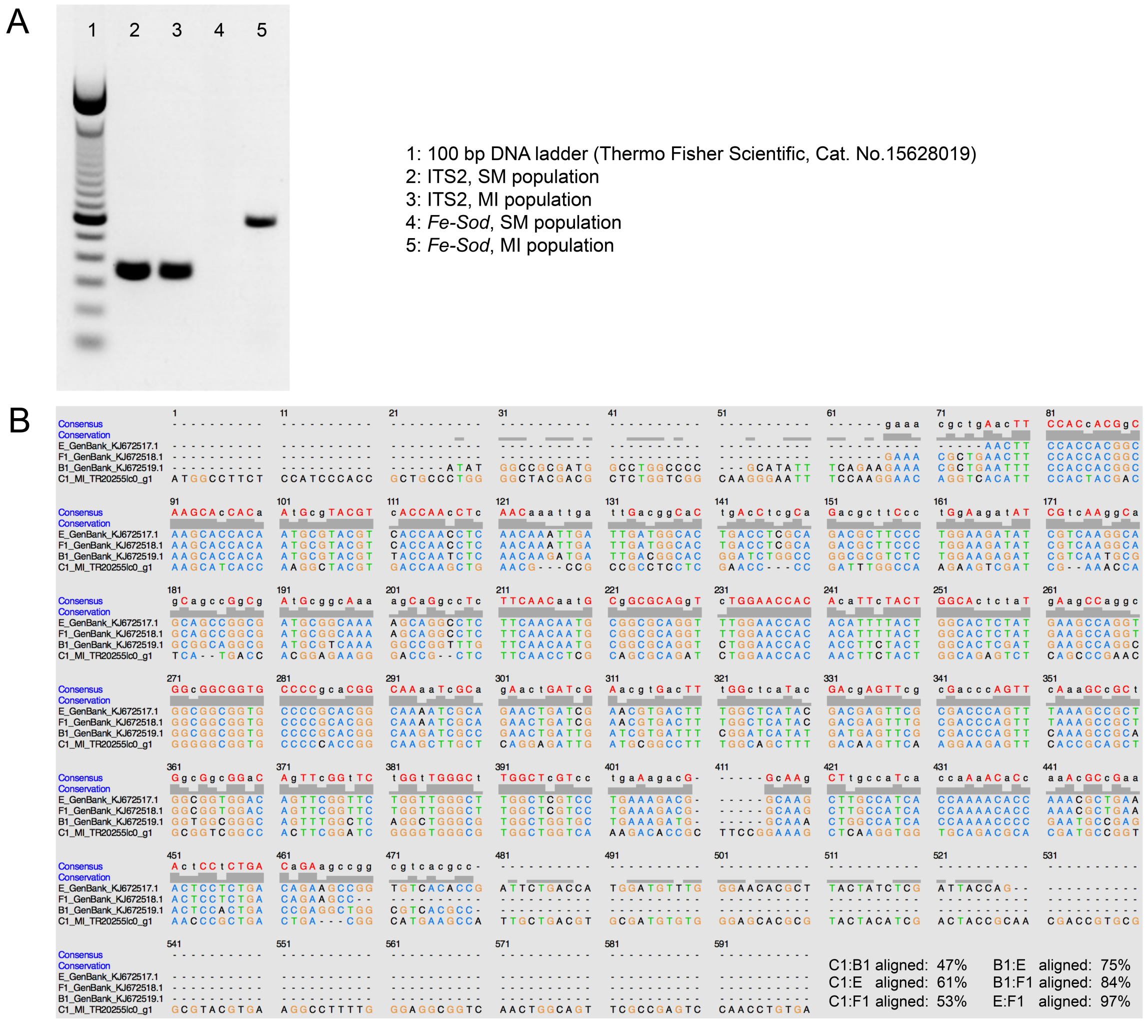

Figure S3.

Presence and sequence similarity of a *Fe-Sod* gene in type C1 *Symbiodinium*.

(A) ITS2 and *Fe-Sod* PCR products amplified from purified SM or MI genomic DNA were imaged after electrophoresis in a 1% agarose gel. (B) Nucleotide sequence alignment with ClustalW (Thompson et al. 2002) revealed 47-61% identity between a full-length *Fe-Sod* gene from the MI population (TR20255|c0_g1, open reading frame: 674-78[-]) and partial-length *Fe-Sod* genes identified by Krueger et al. (2015) in types B1, E, and F1 *Symbiodinium*. Alignments were visualized with UCSF Chimera (Pettersen et al. 2004). Consensus bases across all sequences are red and capitalized. The degree of conservation for each base across all sequences is represented by grey bar height.

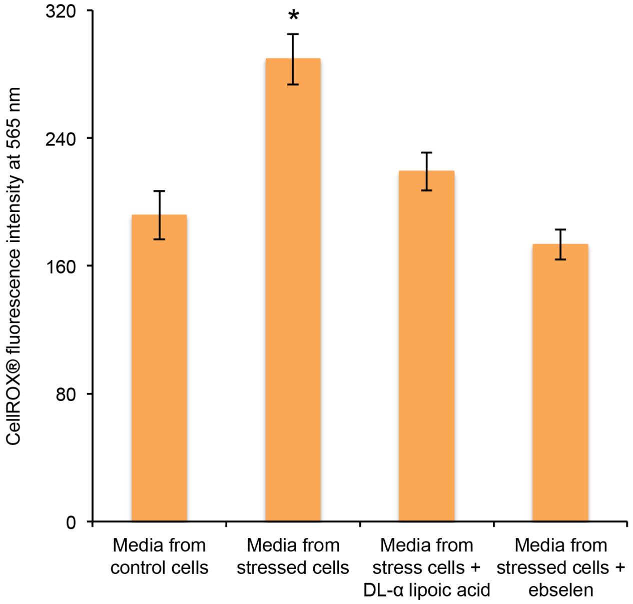

Figure S4.
Validation of CellROX® Orange reagent usage with *Symbiodinium* culture media.

The SM population was kept at 27°C for six days with a 12:12 hour light:dark cycle (50 µmol quanta m^{-2} s^{-1}) for control samples or heated at 32°C for six days with constant light (100 µmol quanta m^{-2} s^{-1}) for stressed samples. 2 mM DL-α lipoic acid (Calbiochem) or 100 µM ebselen (Sigma) was added to samples one hour before incubating media with CellROX® reagent. Asterisks indicate statistically significant (ANOVA) differences between experimental samples (stressed with or without ROS inhibitors; mean ± s.e.m, n = 3) and control samples (mean ± s.e.m, n = 3) at $p < 0.05$.

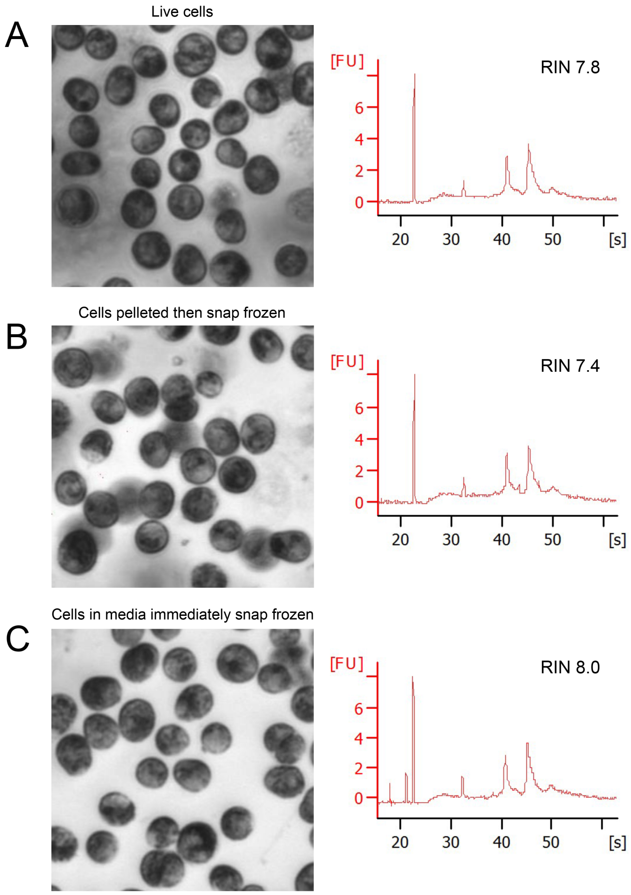

Figure S5.
No detected degradation of *Symbiodinium* immediately snap frozen in media.

Cells from the SM population were imaged on an Olympus fv1000 confocal microscope with the 60x objective, and extracted RNA was run on an Agilent 2100 bioanalyzer. (A) Live cells and a representative bioanalyzer report of RNA integrity. (B) Thawed cells from pelleting culture aliquots by centrifugation, removing media, and then snap freezing cells (~10 minute process) and a representative bioanalyzer report of RNA integrity. (C) Thawed cells that had been immediately snap frozen in media (within 10 seconds of removal from the experimental incubator) and a representative bioanalyzer report of RNA integrity. No sign of cell lysis or loss of RNA integrity between RNA preservation methods was observed.
